# Highly Efficient Polarization-Insensitive Wide-Angle Orthogonal Dipole Metasurface for Ambient Energy Harvesting

**DOI:** 10.3390/mi17050563

**Published:** 2026-05-01

**Authors:** Yiqing Wei, Zhensen Gao, Haixia Li, Zhibin Li

**Affiliations:** 1 Department of Electronics, Xinzhou Normal University, Xinzhou 034000, China; lihaixia0312@163.com (H.L.); lizhibinmailbox@163.com (Z.L.); 2 Intelligent Perception and Fault Prediction Engineering Center, Xinzhou Normal University, Xinzhou 034000, China; 3 School of Information Engineering, Guangdong University of Technology, Guangzhou 510006, China; gaozhensen@gdut.edu.cn

**Keywords:** energy harvesting, metasurface array, polarization-insensitive, rectifier, wide-angle incidence

## Abstract

This work proposes a polarization-insensitive scalable wide-angle metasurface array for highly efficient ambient energy harvesting in the 5.8 GHz Wi-Fi band. Inspired by dipole antenna principles, we design an asymmetric planar orthogonal dipole-based metasurface featuring monolithic integration of Schottky diodes (HSMS-2860) at unit cell feed gaps. This novel direct-impedance-matching strategy eliminates conventional matching networks, reducing energy conversion losses while enabling 99% radiation-to-AC efficiency across all polarization angles at 5.8 GHz. The coplanar architecture interconnects metasurface unit cells via inductors, simultaneously establishing low-loss DC channels and suppressing RF leakage. Fabricated as a 5 × 5 array, the prototype achieves 77.9% peak RF-to-DC efficiency with polarization insensitivity at an incident power of 25 dBm. Furthermore, with incident powers of 15 dBm and 20 dBm, the proposed metasurface array attained RF-to-DC conversion efficiencies exceeding 40% and 60%, respectively. This result indicates that the array is capable of achieving high energy harvesting efficiency across a broad power range. This scalable, drill-free, and polarization-insensitive design demonstrates strong potential for harvesting ambient RF energy in real-world multipath environments.

## 1. Introduction

Metamaterials are artificial media with unique electromagnetic (EM) properties of negative permeability and negative permittivity. It has a promising application in EM cloaking [1,2], anechoic chamber design, biochemical detection [3,4], and radar cross-section reduction [5,6,7]. These studies are based on the principle of the EM absorber, which can reduce reflection by absorbing incoming energy and dissipating it as ohmic losses within the medium [8,9,10]. Although the purpose of the study is achieved, the absorbed energy is also dissipated without being utilized.

Recently, metamaterial absorbers combined with rectifiers for energy harvesting have been widely investigated [11,12,13,14]. Unlike metamaterial absorbers, the metamaterial energy harvester is capable of transferring the absorbed energy to a rectifier, thereby enabling the conversion of RF energy into a storable DC form. However, the available power density in the environment is usually low, which brings difficulties for EM energy harvesting [15]. To overcome this limitation, one method is to expand towards multi-domain energy harvesting [16,17,18,19]. Another method is to improve the efficiency of EM energy harvesting through other approaches. For example, an RF combining structure is proposed for the energy conversion of metasurface arrays [20,21,22]. This structure facilitates the transmission of AC energy, harvested from the entire metasurface array, to the rectifier circuit by establishing a feedback network layer. Such an approach increases the incident power density of the rectifier diode, which in turn extends the diode turn-on time and therefore maximizes the overall power transfer to the load. However, achieving seamless integration between metasurface arrays and radio frequency-integrated composite structures remains a novel challenge. A typical integration approach involves drilling holes in metasurface units [23,24,25]. In this way, the transfer and accumulation of energy from each unit can be achieved, thereby enhancing energy harvesting efficiency. In addition, another way of energy transfer via EM coupling between multilayer metamaterial structures has also been proposed [26]. Similar to the perforation for energy transfer, the feedback network layer is also attached underneath the metasurface array. It is worth noting that while both methods can be effective in harvesting energy, they all require additional layers to construct the feedback network layer. This greatly increases the complexity and fabrication difficulty of metamaterial energy harvesters. Moreover, the added layers will result in energy loss, whether by perforation or EM coupling. For avoiding the energy loss caused by multi-layer structures, a way to integrate the RF combining structure with the metasurface array coplanarity is proposed [27,28,29,30,31,32]. By using the built-in channels of the array to aggregate the collected power from multiple units, the incident power of the rectifier is thus increased. However, the impedance matching of the rectifier circuit is easily influenced by the number of array units, which leads to a decrease in collection efficiency. Furthermore, the energy loss of the components in the rectifier circuit is also a factor that cannot be ignored.

Compared with previous works, the proposed metasurface offers several distinct advantages. First, it demonstrates superior RF-to-DC conversion efficiency specifically tailored for the ubiquitous 5.8 GHz Wi-Fi band, ensuring broad applicability for commercial WPT systems. Second, the design exhibits robust polarization-insensitivity and wide-angle stability, maintaining high harvesting performance across various polarization and incidence angles, which is crucial for operation in complex environments. Third, the metasurface sustains high efficiency over a broad dynamic range of incident power densities, ensuring reliability under fluctuating conditions. Fourth, by integrating the rectifying diodes directly into each unit cell, the design eliminates the need for complex external impedance matching networks and discrete rectifiers. This monolithic integration significantly reduces parasitic and ohmic losses, yielding higher overall energy conversion efficiency than conventional approaches. Finally, the simplified unit cell structure facilitates scalable array expansion, making the design highly adaptable to space-constrained applications.

## 2. Metasurface Array Analysis and Design

**Figure 1:**
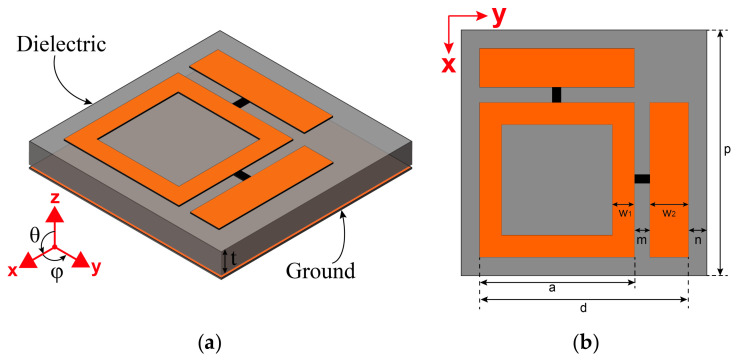
Schematic of the proposed orthogonal dipole metasurface unit cell: (**a**) 3D view, (**b**) top view.

Metasurface absorbers achieve near-perfect EM energy absorption at resonant frequencies by dissipating incident waves through resistive loads [33]. Thus, optimizing energy transfer efficiency to these resistive loads becomes crucial for EM energy harvesting in such absorbers. The proposed metasurface absorber employs a typical sandwich configuration, incorporating a low-loss F4B dielectric layer with optimized thickness t = 3 mm. Both the top patterned resonator and the bottom ground are constructed from copper layers of 35 μm thickness. The top-layer resonant structure evolves from two orthogonally arranged asymmetric planar dipoles, as illustrated in Figure 1. One arm originates from a square frame structure with the middle square patch removed, while the complementary arm comprises a rectangular strip. A resistive load is integrated at the gap of this orthogonal dipole configuration, ensuring that the absorbed EM energy is primarily dissipated through ohmic loss in the load. The F4B dielectric layer exhibits a relative permittivity ε_r_ = 2.2 and an ultra-low loss tangent tanδ = 0.0009. Such minimal dielectric loss renders substrate energy dissipation negligible, making it ideal for metasurface-based energy harvesting applications. The dimensions of the metasurface unit cell are designed to be 14.5 mm × 14.5 mm, which allows it to absorb energy perfectly at 5.8 GHz. The critical geometric parameters of the orthogonally coupled dipole resonator include: a square frame structure with side length *a* = 8 mm and width *w*_1_ = 1 mm, along with a complementary rectangular arm of length *a* = 8 mm (equal to the frame side) and width *w*_2_ = 2 mm. The overall dipole configuration occupies a *d*_2_ = 13 mm × 13 mm area within the metasurface unit cell, maintaining a uniform boundary clearance of *n* = 0.75 mm to adjacent units. An energy harvesting port of width *m* = 1 mm is integrated at the inter-arm gap where the resistive load is positioned. This topology enables simultaneous electric and magnetic resonance coupling to incident waves, while the resistive load maximizes power transfer efficiency to the harvesting port.

The proposed metasurface array was simulated and optimized in CST Microwave Studio, a commercial EM solver. Under periodic boundary conditions, a Floquet port excited the unit cell with a plane wave propagating along the −z-direction. A lumped resistor, integrated at the orthogonal dipole gap, emulates the input impedance of the rectifier. The Schottky diode HSMS2860 was selected as the rectifier due to its low turn-on voltage of 0.28 V and rapid switching characteristics at 5.8 GHz. To achieve a direct match between the metasurface unit and the rectifier, the structure dimension was optimized such that the output impedance at the feeding gap is conjugated to the input impedance of the rectifier. While the input impedance of the HSMS2860 reaches Z_d_ = 185 + j17 Ω when terminated with a 100 Ω load at 5.8 GHz. Therefore, the load at the feeding gap of the unit cell was chosen to be a lumped resistor of Z = 185 – j17 Ω.

**Figure 2:**
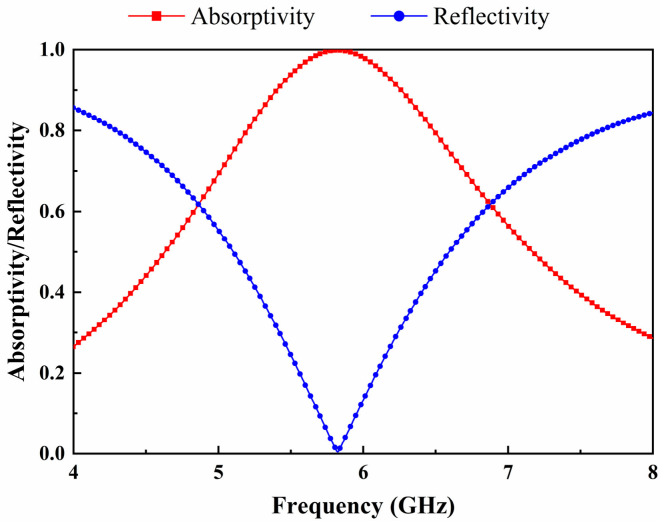
Simulated frequency-dependent absorptivity and reflectivity spectra of the metasurface absorber under normal incidence.

Figure 2 shows the reflectivity and absorptivity of the metasurface unit cell when it is matched to the diode impedance. The absorptivity of the metasurface unit can be expressed as:

(1) $$A\left(\omega \right)=1-R\left(\omega \right)-T\left(\omega \right)=1-{\left|S_{11}\left(\omega \right)\right|}^{2}-{\left|S_{21}\left(\omega \right)\right|}^{2}$$

where $R\left(\omega \right)$ is the reflectivity of the unit cell and $T\left(\omega \right)$ is the transmissivity. The copper ground plane, with thickness (0.035 mm) exceeding three times the skin depth at 5.8 GHz, completely suppresses EM wave transmission. As depicted in Figure 2, the reflectivity of the metasurface unit cell approaches zero at the design frequency of 5.8 GHz, thereby indicating near-unity absorptivity. It confirms that the metasurface unit cell achieves near-perfect absorption at 5.8 GHz when terminated by a conjugate-matched load at the feeding gap, thereby directing nearly all incident energy toward the rectifying circuit.

Next, the input impedance of the metasurface unit cell is analyzed when the load of Z = 185 – j17 Ω is terminated at the feeding gap of the structure. The normalized impedance of the metasurface unit cell can be expressed as follows:

(2) $$Z=\pm \sqrt{\frac{{\left(1+S_{11}(\omega )\right)}^{2}-S_{21}^{2}(\omega )}{{\left(1-S_{11}(\omega )\right)}^{2}-S_{21}^{2}(\omega )}}$$

**Figure 3:**
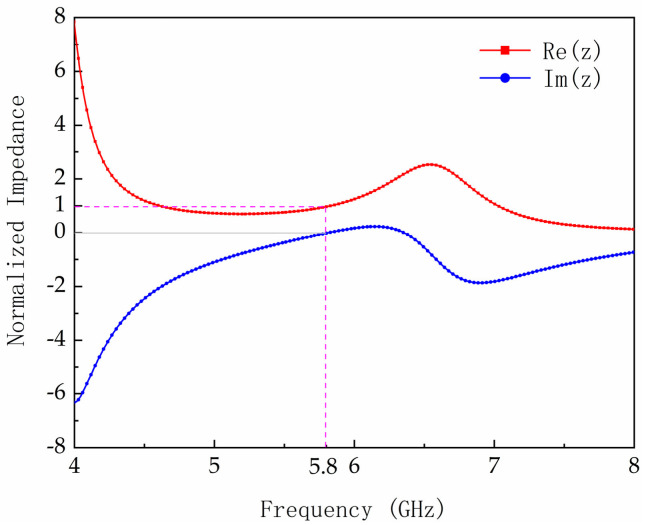
Simulated real [Re(Z)] and imaginary [Im(Z)] parts of the normalized impedance of the metasurface unit cell.

Figure 3 presents the normalized impedance of the metasurface unit cell under conjugate-matched conditions to the rectifying diode. At 5.8 GHz, the real component approaches unity while the imaginary component converges near zero. The impedance matching to free space ensures that nearly all incident EM energy is dissipated in the resistive load, achieving near-perfect absorption for efficient EM energy harvesting.

The energy harvesting capability of the metasurface absorber is quantified by its RF-to-AC conversion efficiency, defined as the ratio of available power delivered to the feeding gap versus total incident RF power:

(3) $$\eta_{Rad-ac}=\frac{P_{ac}}{P_{RF}}$$

where $P_{RF}$ denotes the total time-averaged power incident on the orthogonal dipole, and $P_{ac}$ represents the available time- averaged power received by the load at the feeding gap.

**Figure 4:**
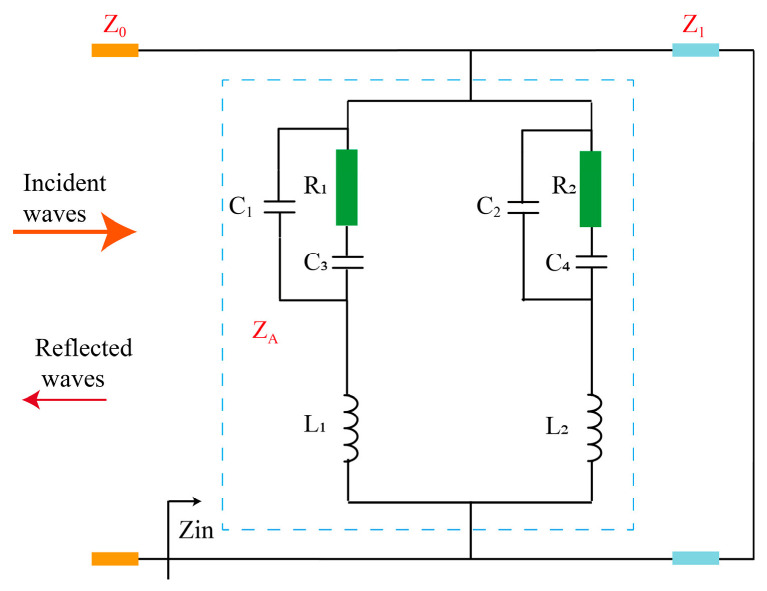
Equivalent circuit model of the metasurface orthogonal dipole unit cell loaded with rectifying diodes.

The circuit-based equivalent modeling approach has been widely adopted to elucidate the absorption mechanisms of periodic absorbers. In this study, the interaction between an incident plane wave and the proposed metasurface array is modeled using a transmission-line equivalent circuit, as illustrated in Figure 4. The metasurface unit cell is equivalent to a resonant LC circuit, with its resonant frequency determined by the values of L and C. The F4B substrate (relative permittivity ε_r_ = 2.2, thickness t = 3 mm) is modeled as a transmission line with a characteristic impedance Z_T_ = 254 Ω, while the ground plane at the back is represented as a short-circuit termination. This LC resonant circuit is terminated by a transmission line representing free space, characterized by an intrinsic impedance of 377 Ω. Rectifying diodes (HSMS2860) are integrated at the gaps in both the x and y directions of the unit cell, respectively. The symbol Z_A_ denotes the combined equivalent impedance of the metasurface resonant element and the integrated rectifying diode. R_1_ and C_3_, along with R_2_ and C_4_, represent the lumped parameters of the diode impedances connected across the feeding ports in the two orthogonal directions. C_1_ and C_2_ denote the inter-gap capacitances, whereas L_1_ and L_2_ correspond to the inductances of the orthogonal dipole patches. While the LC resonant circuit equivalent to the orthogonal dipoles primarily serves to tune the resonant frequency of the metasurface—allowing different structural parameters to adjust the resonant frequency—the input impedance of the rectifying diode, with its nonlinear characteristics, is the critical factor determining the metasurface’s ability to effectively rectify and harvest energy. Optimal energy harvesting efficiency is achieved only when the input impedance of the rectifier is well-matched to the metasurface unit cell at the resonant frequency.

**Figure 5:**
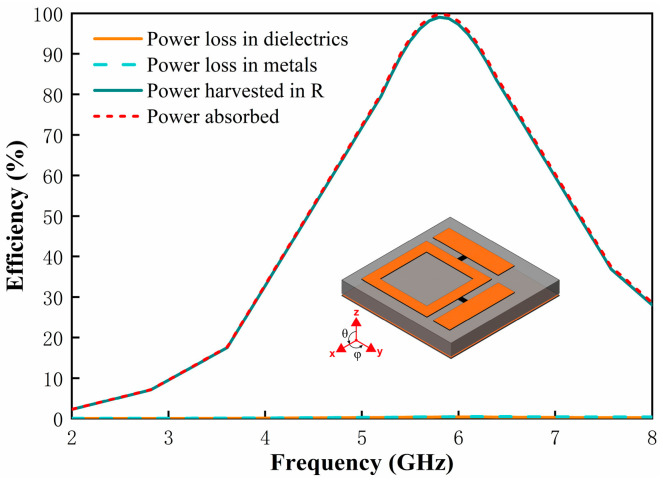
Simulated absorbed power, dissipated power, and harvesting efficiency of the metasurface unit cell under −z-direction plane wave incidence.

Figure 5 depicts the power distribution within the metasurface unit cell under plane wave illumination along the −z-direction with φ = 0° polarization. At 5.8 GHz, near-perfect absorption is achieved (99.8% of incident power), with the absorbed energy partitioned into three components: dissipation in the metallic layers, dielectric loss in the F4B substrate, and power dissipation in the resistive load at the feeding gap. Furthermore, the energy absorbed by the metasurface unit is mainly concentrated at the load of the feeding gap, with a collection efficiency of 99%. While the energy loss on the metal layer and the dielectric layer is almost negligible. This efficient energy channeling stems from the ultra-low loss tangent of F4B (tanδ = 0.0009) and continious copper ground, enabling near-unity power transfer to the harvesting port.

**Figure 6:**
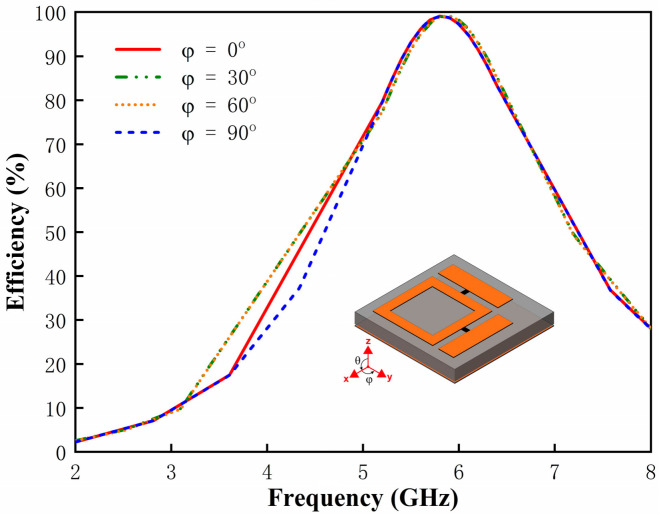
Simulated RF-to-AC power conversion efficiency of the metasurface unit cell under different polarization states.

Subsequently, we evaluated the polarization-insensitive harvesting capability of the metasurface absorber under normal incidence. As illustrated in Figure 6, the RF-to-AC conversion efficiency of the metasurface unit cell exhibits consistency at different polarizations (φ = 0°, 30°, 60°, and 90°). The structure achieves a near-unity efficiency of 99% at 5.8 GHz, demonstrating the independence from the incident electric field polarization.

## 3. Multiphysics and Multiparameter Analysis and Optimization

**Figure 7:**
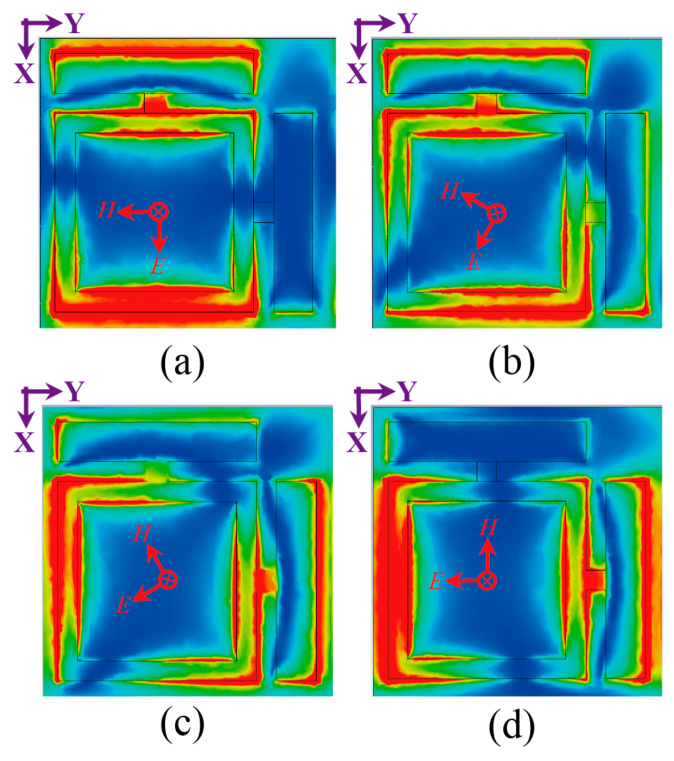
Simulated electric field distributions of the metasurface unit cell at 5.8 GHz for polarization angles of (**a**) 0°, (**b**) 30°, (**c**) 60°, and (**d**) 90°, with the incident plane wave propagating along the −z direction. Field intensity scales from 0 V/m (blue) to 6000 V/m (red).

To investigate the EM energy harvesting characteristics of the metasurface absorber, the physical response of the orthogonal dipole unit was analyzed with the incident plane wave was excited at 5.8 GHz. Under −z-direction illumination, the electric field distribution, surface current, and power flow were examined at φ = 0°, 30°, 60°, and 90°. As depicted in Figure 7a,d, for φ = 0° and φ = 90° polarizations, the electric field predominantly localizes at the feeding gap aligned with the incident electric field direction. In contrast, under oblique polarization incidences, the field distributes across both orthogonal feeding gaps, as shown in Figure 7b,c.

**Figure 8:**
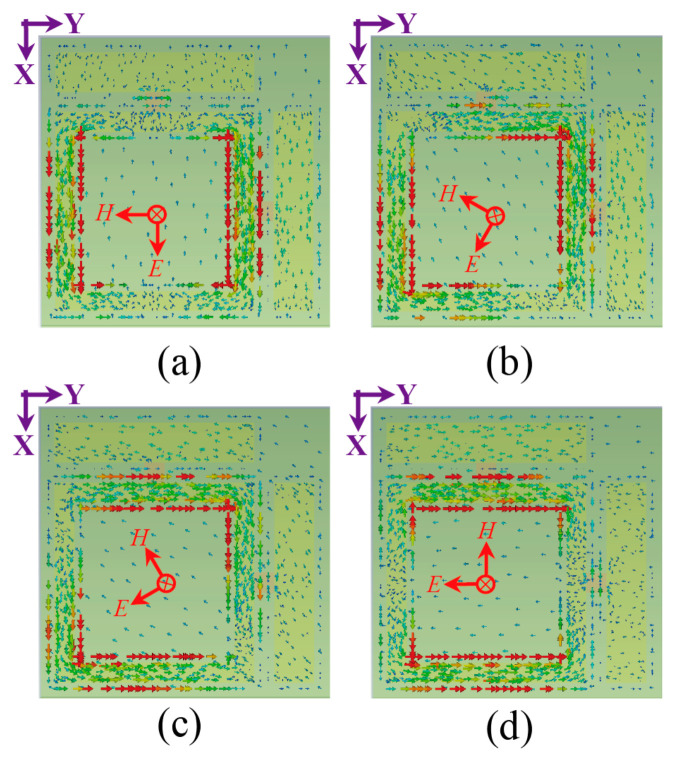
Simulated surface current distributions on the metasurface unit cell at 5.8 GHz for linear polarization angles of (**a**) 0°, (**b**) 30°, (**c**) 60°, and (**d**) 90°, with the incident plane wave propagating along the −z direction. Color scale: 0 to 50 A/m, ranging from blue (minimum) to red (maximum).

Figure 8 presents the corresponding surface current distributions. When the electric field polarization aligns with the dipole axes (φ = 0° or 90°), currents primarily flow along the square ring arm parallel to the incident field, as shown in Figure 8a,d. For oblique polarization states (φ = 30° and 60°), surface current density concentrates predominantly along both orthogonal arms of the crossed dipole structure, as illustrated in Figure 8b,c. It arises from the projection decomposition of incident electric field vectors onto the dipole’s orthogonal axes, enabling simultaneous excitation of coupled electric dipole modes in non-principal directions.

**Figure 9:**
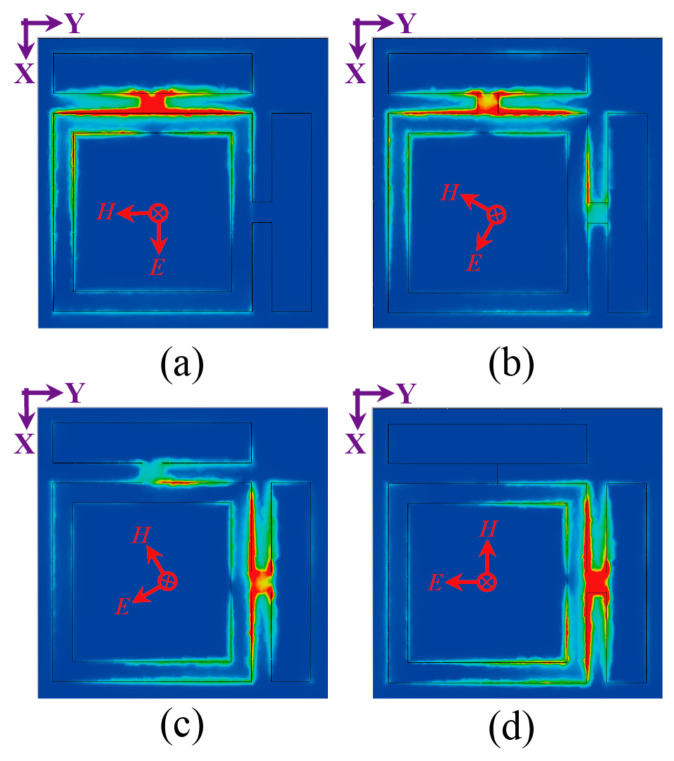
Simulated power flow distributions within the metasurface unit cell at 5.8 GHz for linear polarization angles of (**a**) 0°, (**b**) 30°, (**c**) 60°, and (**d**) 90°, with the incident plane wave propagating along the −z direction. Color scale: 0 to 5 × 10^5^ W/m^2^, ranging from blue (minimum) to red (maximum).

Figure 9 shows the power flow distributions of the orthogonal dipole unist at polarizations of φ = 0°, 30°, 60° and 90°. When φ = 0°, the majority of EM energy localizes at the y-direction feed gap, forming a polarization-matched hotspot aligned with the incident electric field. As φ increases to 30° and 60°, energy redistributes bidirectionally due to projection decomposition of the incident field onto orthogonal axes, generating resonances at two feed gaps onto the orthogonal dipole arms. At φ = 90°, energy relocates to the x-direction feed gap, demonstrating the close correlation between energy aggregation and geometrically symmetric structure.

**Figure 10:**
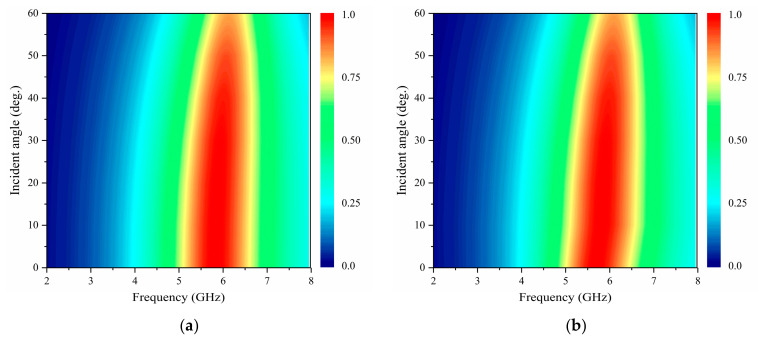
Simulated absorption spectra of the metasurface unit cell as a function of incidence angle θ for: (**a**) TE polarization and (**b**) TM polarization, where θ varies from 0° to 60°.

The sensitivity of the metasurface to both polarization angle and incident angle is a critical factor in evaluating its energy harvesting efficiency. The angular robustness of energy harvesting is quantified in Figure 10, depicting the absorptivity of orthogonal dipole units under TE and TM polarized waves at incident angles from 0° to 60°. At 5.8 GHz, absorptivity exhibits a slight decrease for both polarizations as θ increases from 0° to 60°, yet remains above 0.9 even at θ = 60°. This angular insensitivity primarily stems from geometric symmetry conservation, wherein the rotational symmetry of the orthogonal dipole structure minimizes polarization-dependent losses. While a slight decrease in absorptivity occurs due to diminished EM resonance strength-arising from the reduced intensity of the EM wave’s vertical component-the metasurface maintains robust performance across incident angles.

**Figure 11:**
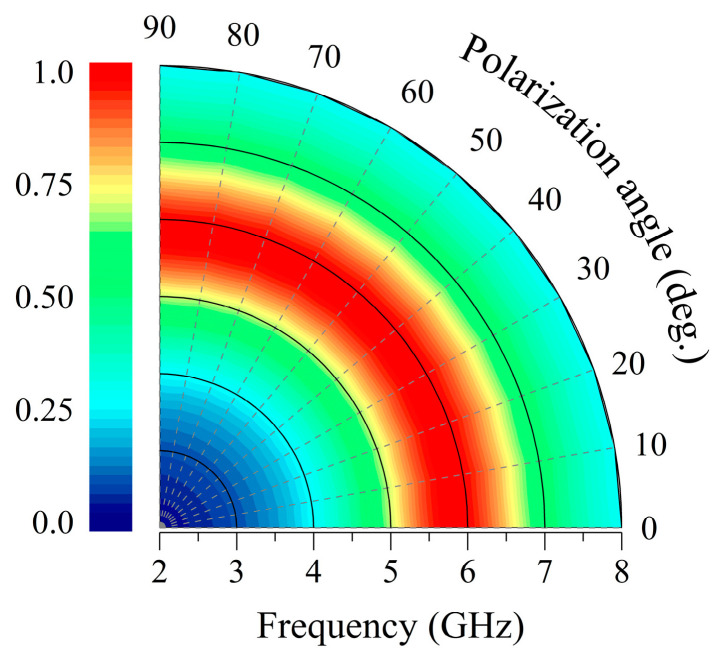
Simulated absorption spectra of the metasurface unit cell under different polarization angles at normal incidence.

Figure 11 quantifies the polarization-robust absorption characteristics of orthogonal dipole units, demonstrating the absorptivity exceeding 0.9 across all polarization angles (φ = 0–90°) at 5.8 GHz. This strict angular independence originates from the unit cell’s rotational symmetry, which enables the incident field vector to be decomposed into two orthogonal components (E_x_ and E_y_). This decomposition ensures identical EM responses to arbitrary linear polarizations by allowing the two dipole branches to operate independently without cross-coupling. Crucially, the polarization-insensitive absorption profile shown in Figure 10 exhibits the congruence with the angularly stable energy harvesting efficiency presented in Figure 6, validating that: (1) geometric invariance dominates: the symmetric topology maintains constant effective inductance and capacitance regardless of the polarization angle, thereby eliminating resonance detuning and preserving the fidelity of impedance matching; and (2) polarization insensitivity enables decoupled omnidirectional energy harvesting, allowing the structure to robustly collect energy in complex EM environments where the incident polarization state is arbitrary.

**Figure 12:**
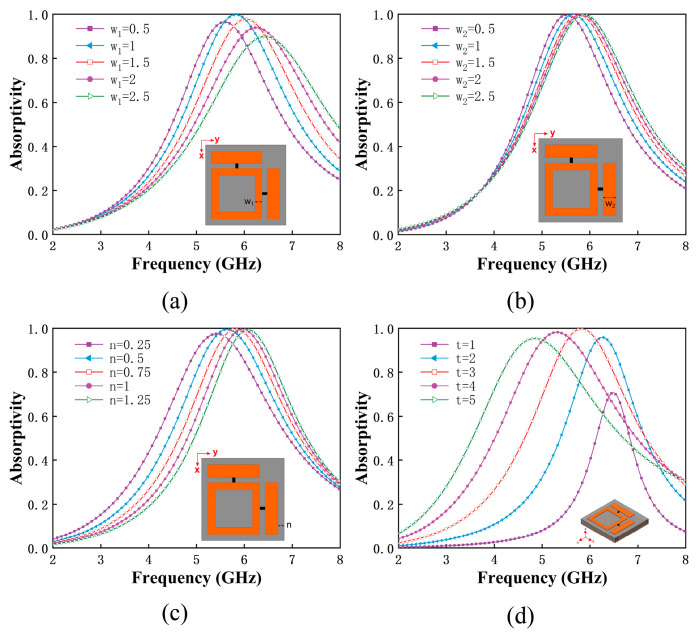
Simulated absorptvity of the metasurface unit cell at normal incidence under TE polarization as a function of key structural parameters: (**a**) square ring width *w*_1_, (**b**) rectangular width *w*_2_, (**c**) boundary gap *n*, (**d**) dielectric layer thickness *t*.

To further investigate the absorption properties of orthogonal dipole metasurface, the absorptivity of dimensional parameters of structural units is analyzed. The influence of the square ring width *w*_1_ on the absorption characteristics is analyzed, maintaining a constant outer ring edge length a where *w*_1_ is complementary to the inner ring dimension, as shown in Figure 12a. Increasing *w*_1_ induces a distinct blueshift in the absorption peak of the orthogonal dipole units. While the absorptivity remains above 0.9 across the tested range, significant spectral shifts occur. The phenomenon arises because variations in *w*_1_ alter the equivalent inductance and capacitance of the unit cell, functioning as an LC resonator. Consequently, the resonant frequency shifts, and impedance mismatch develops between the load at the gap and the metasurface unit cell, ultimately reducing absorption efficiency.

The absorption response dependence on the rectangular arm width *w*_2_ is presented in Figure 12b. As *w*_2_ increases, theabsorption peak undergoes a blueshift, while the absorptivity remains essentially unchanged, close to 1. This frequency shift results from a reduction in the equivalent inductance of the dipole arm as *w*_2_ widens, thereby increasing the resonance frequency. Crucially, variations in *w*_2_ within a limited range primarily affect the resonant frequency without degrading the absorptivity. This tunability allows for the selection of an optimal *w*_2_ to achieve near-perfect EM energy absorption at specific target frequencies, facilitating frequency-agile designs. The impact of the orthogonal dipole boundary gap size *n*, equivalent to half the inter-unit cell spacing, on absorption performance is shown in Figure 12c. An increase in *n* induces a blueshift in the absorption peak, with the absorptivity consistently exceeding 0.9. It demonstrates that the mutual EM coupling between adjacent structural units significantly influences the resonant behavior of the individual unit cell. The observed dependence underscores the non-negligible role of near-field interactions within the metasurface array on the absorption properties.

Figure 12d depicts the absorptivity variation with dielectric substrate thickness *t*. Increasing *t* induces a redshift in the absorption peak, accompanied by a significant modification in absorptivity and a progressive broadening of the absorption bandwidth. The absorptivity of the metasurface unit approaches 1 and reaches a maximum at a thickness *t* of 3 mm. These pronounced modifications confirm that dielectric thickness governs both the spectral selectivity (peak position) and energy capture capacity (bandwidth and magnitude) of the metasurface. Optimal thickness selection is therefore essential for maximizing energy harvesting efficiency across desired frequency bands.

Subsequently, the lumped resistive load at the orthogonal dipole gap was replaced with the rectifier diode HSMS2860. Simulation results demonstrate that conjugate impedance matching between the rectifier diode and the orthogonal dipole eliminates the need for an external matching network, thereby simplifying the RF-to-DC conversion efficiency. These metasurface units are interconnected via integrated inductors serving dual roles: (1) as an AC filter suppressing RF signal leakage, and (2) as DC current channels enabling parallel power combining. It avoids the construction of energy flow channels by drilling holes, thus simplifying the structural layout of the metasurface.

**Figure 13:**
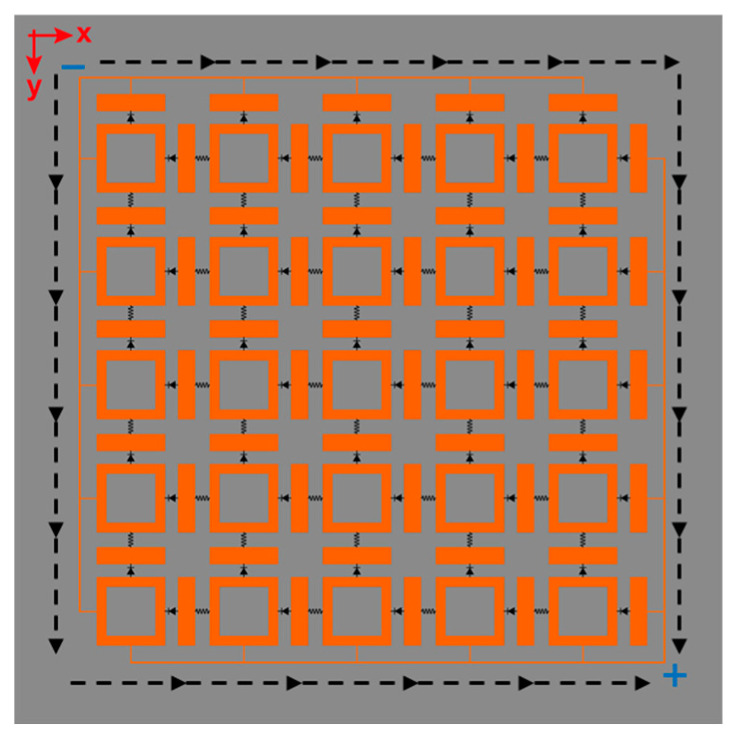
Schematic diagram of the DC channel topology for the me tasurface energy harvester.

Figure 13 schematically illustrates the DC channel topology. Using a 5 × 5 array of orthogonal dipole units, adjacent units are interconnected via inductors. Each row and column terminates at integrated microstrip lines for consolidated energy harvesting. For incident electric field polarization at φ = 0°, diodes within each y-axis column rectify the RF signal, generating current flow along the y-axis. Inductors interconnect units within each column, channeling vertical current towards the terminal microstrip lines for column-wise power aggregation. Consequently, the power harvested by each column transfers to the array terminal. Conversely, under φ = 90° polarization, diodes rectify the signal along the x-axis, inducing horizontal current flow. Similarly, inductors induce the current within each row along the x-axis to the terminal lines, aggregating row-wise power at the array terminal. Crucially, for arbitrary polarization angles (0° < φ < 90°), both diodes within each unit cell co-rectify the RF signal. Resultant currents flow simultaneously along both orthogonal arms. These currents propagate via the x and y-axis inductor networks to the terminal microstrip lines, achieving power aggregation at the array terminal. This metasurface array topology thus achieves robust energy transfer and aggregation independent of the incident E-field polarization direction.

## 4. Measurement and Discussion

**Figure 14:**
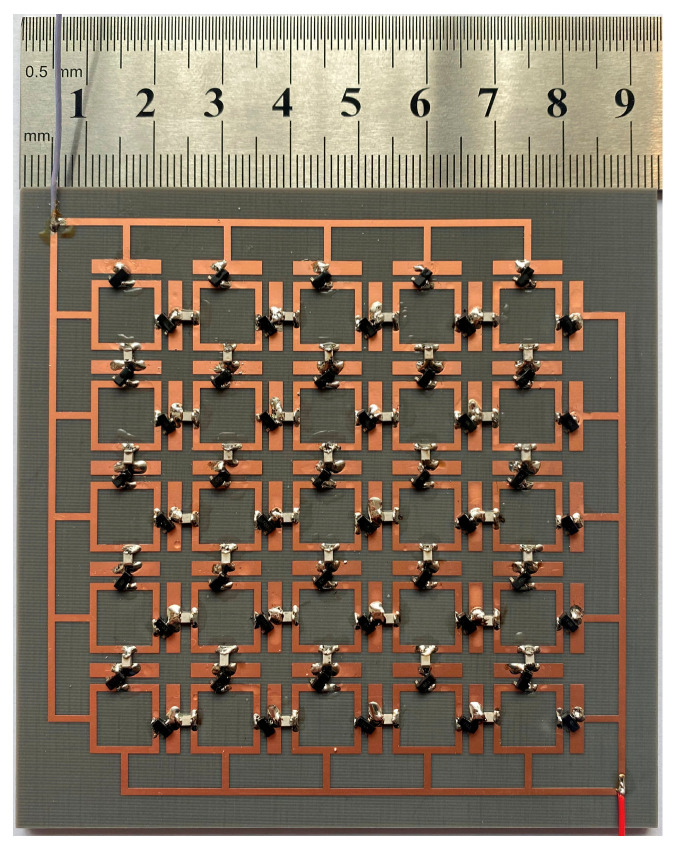
Fabricated prototype of the metasurface energy harvester incorporating integrated Schottky diode rectifiers.

A 5 × 5 orthogonal dipole metasurface energy harvester was fabricated as shown in Figure 14. The fabricated prototype was implemented on a 3 mm thick F4B dielectric substrate (ε_r_ = 2.2, tanδ = 0.0009) with 35 μm copper cladding forming both the toplayer dipole array and bottom ground plane. The dimensions of the entire array occupy an area of 72.5 mm × 72.5 mm. Each unit cell integrates Schottky barrier diodes (Avago HSMS-2860) at feed gaps for rectification, while adjacent units are interconnected via 47-nH inductors that simultaneously block RF leakage and establish a low-loss DC channel.

**Figure 15:**
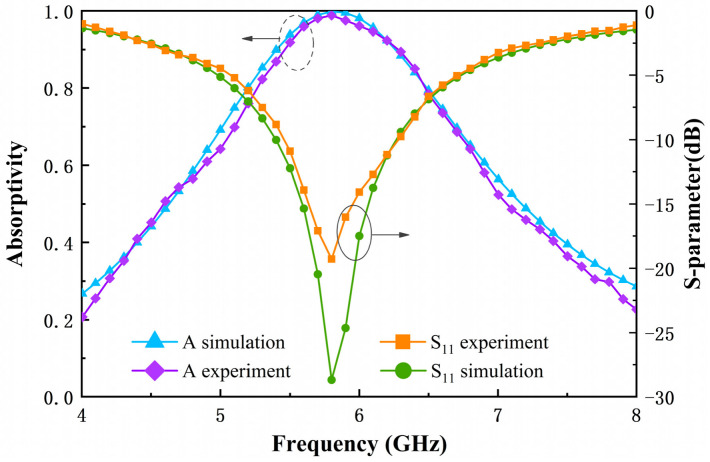
Comparison between measured and simulated reflection spectra and absorption of the designed metasurface array under normal incidence.

To validate the reflection and absorption performance of the designed metasurface array, a bow-frame method was employed for absorption measurement. Figure 15 presents a comparison between the simulated and measured reflection and absorption spectra under normal incidence. The experimental results demonstrate good agreement with the simulation, confirming the effectiveness of the design. The minor frequency shifts and variations in resonance depth are attributed to experimental uncertainties, fabrication tolerances, and parasitic effects introduced during the soldering of lumped elements.

**Figure 16:**
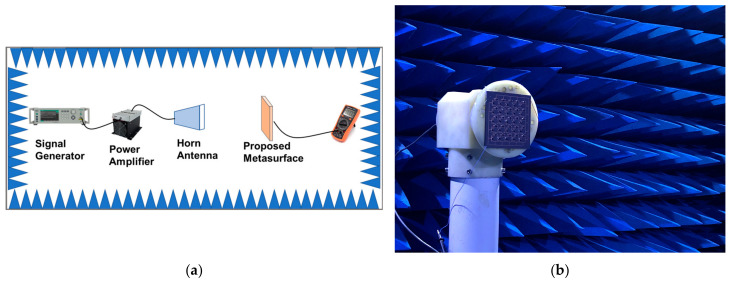
(**a**) Schematic experimental setup and (**b**) measurement photograph for characterizing metasurface array energy harvesting in an anechoic chamber.

The configuration of the measurement setup for the DC energy harvesting platform is shown in Figure 16. Incident plane waves were generated by a signal generator, amplified through a power amplifier, and radiated toward the prototype via a standard-gain horn antenna covering the operational band. The prototype was positioned 1 m away in the far field. DC output voltage was then measured across the load terminals using a digital multimeter. The RF-to-DC collection efficiency of the metasurface array can be expressed as follows [33,34]:

(4) $$\eta_{Rad-dc}=\frac{P_{dc}}{P_{in}}$$

where *P*_dc_ is the total DC power received by the array, which can be calculated as follows:

(5) $$P_{dc}=\frac{V_{out}^{2}}{R_{load}}$$

where *V*_out_ is the dc voltage across the load *R*_load_ as recorded by the digital multimeter. The total available incident power *P*_in_ to the metasurface array equals the product of the antenna-radiated power density at the prototype and the array surface area:

(6) $$P_{in}=\frac{G_{t}\cdot P}{{4\pi R}^{2}}\cdot A_{s}$$

where *A*_s_ denotes the effective receiving aperture of the metasurface array-typically approximated as its physical surface area [20,35,36]. Here, *P* represents the transmitter output power from the signal generator, *G_t_* is the horn antenna gain, and *R* represents the distance from the horn antenna to the prototype.

The efficiency calculation is based on an incident-power model using the physical area of the array. It is important to note that this approach assumes uniform illumination across the entire array aperture. In practice, slight non-uniformities in the incident field may exist due to the finite distance between the transmitting antenna and the array. Additionally, the effective aperture approximation assumes that the entire physical area contributes equally to energy harvesting, which may not fully account for edge effects or near-field coupling. To ensure repeatability, all measurements were conducted multiple times under identical conditions.

**Figure 17:**
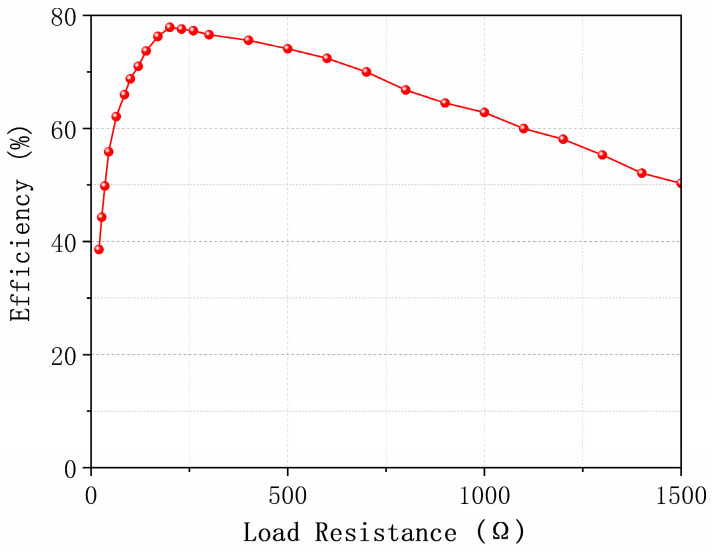
Measured RF-to-DC conversion efficiency of the metasurface energy harvester versus load resistance under normal incidence at 5.8 GHz with an incident power of 25 dBm.

Figure 17 demonstrates the RF-to-DC conversion efficiency of the metasurface array as a function of load impedance under normal-incidence plane wave irradiation. Maximum rectification efficiency occurs at 5.8 GHz with a 200 Ω load resistance. This optimal impedance aligns with the simulation results indicating conjugate matching at 100 Ω per dipole-rectifier pair. The observed 200 Ω impedance results from two series-connected rectifier HSMS-2860 diodes terminating opposite feeding gaps of each unit cell, thereby doubling the individual diode impedance. The efficiency curve exhibits characteristic nonlinear behavior, showing a rapid increase as the load resistance approaches 200 Ω and reaching a peak efficiency of 77.9% at this point. Beyond 250 Ω, efficiency declines significantly due to power reflection caused by impedance mismatch.

**Figure 18:**
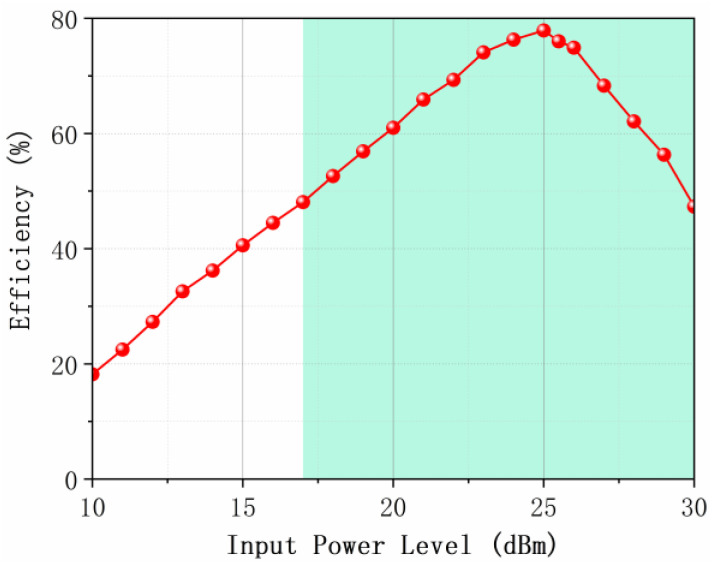
Measured RF-to-DC conversion efficiency of the metasurface harvester as a function of incident power at 5.8 GHz under normal incidence with a load resistance of 200 Ω.

The input power of the metasurface array was systematically scanned to identify the optimal operating point. Figure 18 quantifies the RF-to-DC conversion efficiency of the array versus incident power under normal-incidence plane wave illumination. The array achieves a peak efficiency of 77.9% at an incident power of 25 dBm, coinciding with conjugate impedance matching between the rectifier diodes (HSMS-2860) and the unit cell. In addition, the efficiency curve exhibits characteristic nonlinear behavior, showing a steady rise from 10 dBm to 25 dBm and peaking at 25 dBm. Beyond 26 dBm, efficiency degrades rapidly owing to impedance mismatch. Furthermore, the metasurface array demonstrates high DC energy conversion efficiency over a wide incident power range.

**Figure 19:**
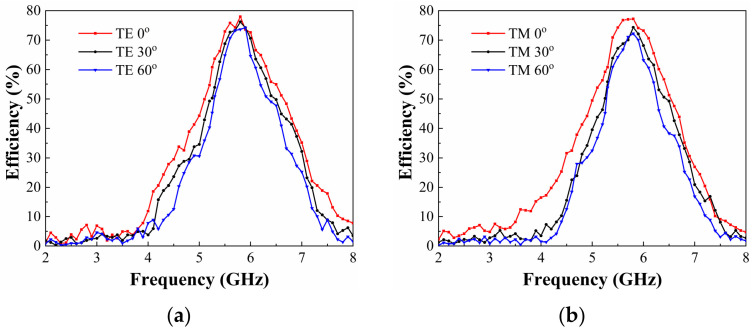
Measured RF-to-DC conversion efficiency of the metasurface array under oblique incidence with an incident power of 25 dBm and a load resistance of 200 Ω: (**a**) TE-polarized wave, (**b**) TM-polarized wave.

Finally, under optimized input power (25 dBm) and load impedance (200 Ω), the RF-to-DC energy collection efficiency was characterized across incidence angles and polarizations. Figure 19 presents the energy harvesting efficiency of the metasurface array across incident angles (0–60°) under TE and TM polarizations. The array exhibits exceptional angular stability with invariant resonant frequency for both polarizations. However, both peak efficiency and half-power bandwidth exhibit angular dependence. Under TE polarization, the harvesting efficiency declines from 77.9% at normal incidence to 74.2% at θ = 60°. For TM polarization, the efficiency decreases to 72.2% at 5.8 GHz when θ = 60°. Corresponding half-power bandwidth reductions occur for both polarizations at 60° incidence. This phenomenon originates from two mechanisms: reduced vertical electric field strength decaying proportionally to cosθ, which weakens resonant excitation, and unit cell illumination area decreases with θ, thereby reducing energy capture cross-section.

**Figure 20:**
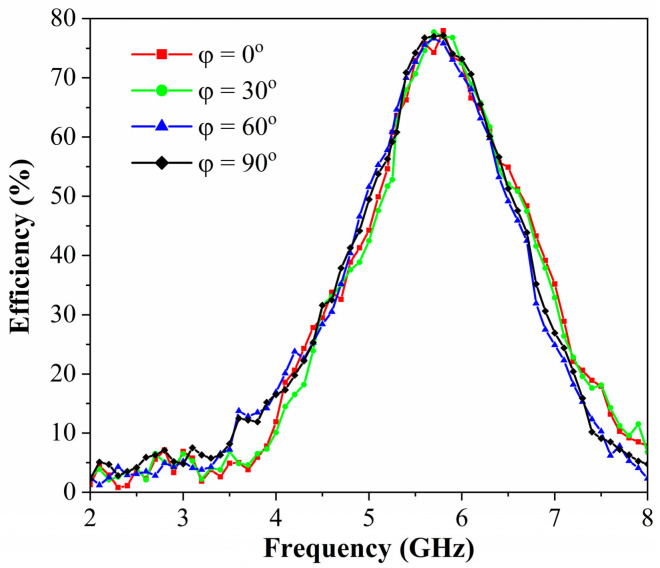
Measured RF-to-DC conversion efficiency spectra of the metasurface harvester versus frequency under different polarization states at 25 dBm incident power with a 200 Ω load.

Figure 20 presents polarization-insensitive energy harvesting efficiency curves of the metasurface array. Measured results demonstrate stable efficiency up to 77.9% at 5.8 GHz across polarization angles, demonstrating near-unity correspondence with simulations in Figure 5. For frequencies beyond 5.8 GHz, significant deviations between measured and simulated results occur due to rectifier diode nonlinearities under varying input power and frequency conditions. Overall, the metasurface array sustains polarization-insensitive operation at 5.8 GHz, maintaining peak harvesting efficiency of 77.9% across all incident angles.

**Figure 21:**
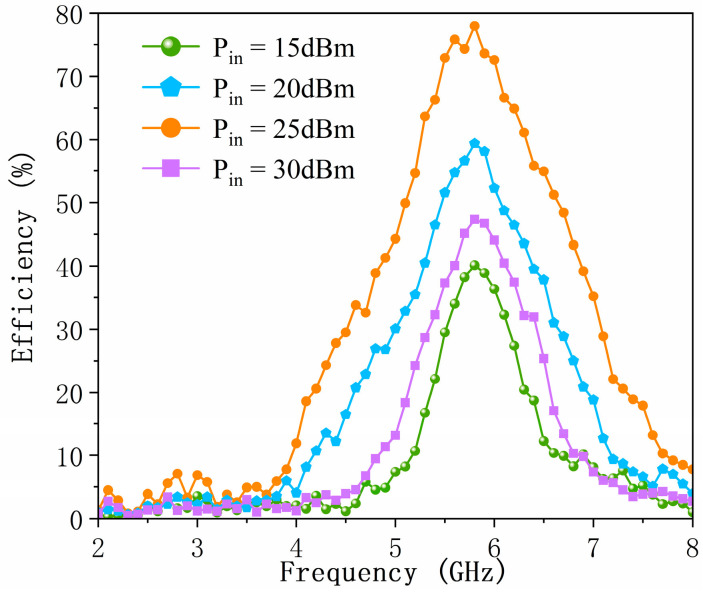
Measured RF-to-DC conversion efficiency versus frequency for the metasurface energy harvester under TE-polarized normal incidence at various incident power levels.

Figure 21 depicts the measured RF-to-DC conversion efficiency as a function of frequency for the proposed metasurface-based energy harvester under TE-polarized normal incidence at different incident power levels. To further evaluate the prototype performance across varying power conditions, the RF-to-DC conversion efficiency was characterized at input power levels of 15 dBm, 20 dBm, 25 dBm, and 30 dBm. Owing to the nonlinear behavior of the rectifying diodes under different excitation levels, frequency conditions, and load impedances, the maximum RF-to-DC conversion efficiencies measured at 15 dBm, 20 dBm, 25 dBm, and 30 dBm are 40.6%, 61.3%, 77.9%, and 47.4%, respectively. These results demonstrate that the proposed energy harvester maintains high conversion efficiency over a wide range of incident power levels. Additionally, the results presented in Figure 19 and Figure 20 verify the wide-angle tolerance and polarization insensitivity of the designed prototype, confirming its robust performance in complex EM environments beyond controlled anechoic chamber conditions.

The observed deviations between the simulated and measured results are primarily attributed to four practical factors. First, fabrication tolerances inherent in the PCB process, such as minor variations in substrate thickness and etching line widths, alter the effective electrical dimensions of the unit cells. Second, parasitic parameters intrinsic to the rectifying diodes (e.g., package lead inductance and pad capacitance) and soldering joints introduce additional impedance that is often neglected in idealized models. Third, finite array effects arise because the fabricated metasurface consists of a limited number of elements, whereas simulations typically assume an infinite periodic structure. The resulting edge effects and mutual coupling deviations contribute to discrepancies in resonance and scattering characteristics. Finally, measurement uncertainties, including RF power meter calibration errors, cable losses, and connector mismatches, induce fluctuations in the measured RF-to-DC conversion efficiency. Despite these factors, the experimental results exhibit excellent qualitative agreement with the simulations in terms of operational trends and bandwidth, thereby validating the robustness of the proposed design.

Table 1 gives a comparison of the proposed metasurface array with other reports on energy harvesting in the literature, demonstrating its capability to harvest EM energy in 5.8 GHz WiFi band. Crucially, our design eliminates impedance matching networks by directly integrating rectifier diodes within the unit cell, thereby maintaining high DC harvesting efficiency across broader frequency bands and input power ranges. Furthermore, the coplanar inter-unit connections simplify the metasurface structure by avoiding via-hole drilling required in conventional designs. Additionally, polarization-insensitive operation ensures stable efficiency across varying polarization angles. Consequently, these innovations yield a fundamentally simplified architecture that achieves matching through direct geometric optimization rather than discrete matching networks, significantly enhancing manufacturability for scalable energy harvesting applications.

## 5. Conclusions

This work demonstrates a polarization-insensitive metasurface array based on orthogonally arranged cross-dipole elements for efficient 5.8 GHz EM energy harvesting. By directly integrating rectifier diodes at two feeding gaps of each unit cell, the design eliminates impedance matching networks while achieving 77.9% peak RF-to-DC efficiency across all polarizations. Surface-mounted inductors interconnect the 5 × 5 array to establish continuous DC current-combining channel, enabling efficient energy transfer to loads through microstrip lines. The coplanar integration of metasurface unit cells, rectifiers, and DC channel significantly reduces fabrication complexity and cost by eliminating through-substrate vias. This design consequently exhibits exceptional scalability and environmental adaptability, providing an optimized solution for harvesting ambient RF energy in multipath-rich scenarios.

## Figures and Tables

**Table 1:** Comparison of the proposed energy harvester meta surface with recently related work.

Reference	Frequency (GHz)	Polarization	Diodes to Each Cell	Dimension of Unit Cell (mm)	Prototype Size (mm)	RF-to-DC Efficiency (%)
[20]	Single-band 2.4	Dual LP	No	70 × 70 × 4.804 (5 layers)	280 × 280 (array: 4 × 4)	70% (9 dBm)
[26]	Single-band 5.8	Polarization Insensitive	No	31.7 × 31.7 (5 layers)	190.2 × 190.2 (array: 6 × 6)	55% (35 μW/cm^2^)
[29]	Dual-band 2.4, 12.6	LP	No	21 × 21 × 3.07 (3 layers)	1822 × 157 (array: 7 × 7)	64%@2.4 GHz (3 dBm), 55%@12.6 GHz (14 dBm)
[30]	Dual-band 2.1, 5.8	Polarization Insensitive	No	36.9 × 36.9 × 3.07 (3 layers)	184.5 × 184.5 (array: 5 × 5)	64.8%, 41.1% (46.4 μW/cm^2^)
[35]	Single-band 2.45	Dual LP	No	74 × 67 × 4.769 (5 layers)	229 × 305 (array: 3 × 4)	61% (313 μW/cm^2^)
[36]	Dual-band 2.4, 5.8	Polarization Insensitive	Yes	16 × 15.5 × 1.27 (3 layers)	67 × 64 (array: 4 × 4)	58%, 51% (0 dBm)
[37]	Single-band 2.84	LP	Yes	50 × 50 × 3.175 (3 layers)	200 × 200 (array: 4 × 4)	60% (187.5 μW/cm^2^)
[38]	Single-band 2.45	Polarization Insensitive	NA	62.4 × 62.4 (6 layers)	124.8 × 124.8 (array: 2 × 2)	62% (175 μW/cm^2^)
[39]	Single-band 2.4	Dual LP	No	25 × 25 (3 layers)	90 × 90 (array: 2 × 2)	55% (0 dBm)
[40]	Single-band 2.45	LP	Yes	20 × 20 × 4.2 (6 layers)	120 × 120 (array: 6 × 6)	66.9% (5000 μW/cm^2^)
This work	Single-band 5.8	Polarization Insensitive	Yes	14.5 × 14.5 × 3.07 (3 layers)	72.5 × 72.5 (array: 5 × 5)	77.9% (25 dBm) 61.3% (20 dBm) 40.6% (15 dBm)

## Data Availability

The original contributions presented in this study are included in the article. Further inquiries can be directed to the corresponding author.
